# Clinical features of myasthenia gravis with neurological and systemic autoimmune diseases

**DOI:** 10.3389/fimmu.2023.1223322

**Published:** 2023-09-14

**Authors:** Ying Zhu, Benqiao Wang, Yuehan Hao, Ruixia Zhu

**Affiliations:** Department of Neurology, The First Affiliated Hospital of China Medical University, Shenyang, China

**Keywords:** autoimmune diseases, myasthenia gravis, neuromyelitis optica, autoimmune encephalitis, multiple sclerosis, inflammatory myopathy

## Abstract

Multiple reports on the co-existence of autoimmune diseases and myasthenia gravis (MG) have raised considerable concern. Therefore, we reviewed autoimmune diseases in MG to explore their clinical presentations and determine whether the presence of autoimmune diseases affects the disease severity and treatment strategies for MG. We reviewed all the major immune-mediated coexisting autoimmune conditions associated with MG. PubMed, Embase and Web of Science were searched for relevant studies from their inception to January 2023. There is a higher frequency of concomitant autoimmune diseases in patients with MG than in the general population with a marked risk in women. Most autoimmune comorbidities are linked to AChR-MG; however, there are few reports of MuSK-MG. Thyroid disorders, systemic lupus erythematosus, and vitiligo are the most common system autoimmune diseases associated with MG. In addition, MG can coexist with neurological autoimmune diseases, such as neuromyelitis optica (NMO), inflammatory myopathy (IM), multiple sclerosis (MS), and autoimmune encephalitis (AE), with NMO being the most common. Autoimmune diseases appear to develop more often in early-onset MG (EOMG). MS coexists more commonly with EOMG, while IM coexists with LOMG. In addition, MG complicated by autoimmune diseases tends to have mild clinical manifestations, and the coexistence of autoimmune diseases does not influence the clinical course of MG. The clinical course of neurological autoimmune diseases is typically severe. Autoimmune diseases occur most often after MG or as a combined abnormality; therefore, timely thymectomy followed by immunotherapy could be effective. In addition, thymoma-associated AChR MG is associated with an increased risk of AE and IM, whereas NMO and MS are associated with thymic hyperplasia. The co-occurrence of MG and autoimmune diseases could be attributed to similar immunological mechanisms with different targets and common genetic factor predisposition. This review provides evidence of the association between MG and several comorbid autoimmune diseases.

## 1 Introduction

Myasthenia gravis (MG) is an antibody-mediated disease with T-cell-driven immune pathogenesis and complex interactions between CD4+ T cells and B cells characterized by fatigable muscle weakness of the ocular, facial, bulbar, respiratory, and limb muscles. Nearly 80-85% of MG cases are associated with acetylcholine receptor antibodies (AChR-Abs) that destroy synaptic transmission across the neuromuscular junction (1). Moreover, other autoantibodies have been identified in patients with MG, including Abs against muscle-specific tyrosine kinase (MuSK) and lipoprotein receptor-related protein 4 (LRP4), which are detected in approximately 5% of MG patients. AChR belongs to complement activation IgG1-3 immunoglobulin G1-complement activating Abs. The pathophysiology of MuSK-MG differs from that of AChR-MG, with non-complement fixing IgG4 as an anti-MuSK Ab. MG shows high heterogeneity in clinical features as well as in the Ab repertoire, and MG is divided into subgroups according to age of onset, autoantibody type, thymus pathology, and clinical manifestations (2–6).

The thymus is affected in most patients with AChR-positive MG; approximately 70% of the patient have thymic hyperplasia, 10% thymoma and the remainder have either a normal or atrophic thymus. The thymus is an important site for T-cell maturation and induces self-tolerance. Thymic dysfunction not only leads to the activation of CD4+ T cells and exhausts T regulatory cells (Tregs). Activated CD4+ T cells can interact with B lymphocytes to produce autoantibodies that attack neuromuscular junctions, disrupt self-tolerance and induce autoimmunity, whereas T helper type 17 (Th17) cells produce the interleukin (IL)-17 and induce inflammation and autoimmunity. IL-12 and IFN-γ produced by Th1 cells and IL-4, IL-6, and IL-10 produced by Th2 cells mediate and generate an inflammatory cascade and Ab production. Th17/Treg imbalance and Th1/Th2 imbalances could be responsible for autoimmune inflammation and are implicated in the pathogenesis of MG (7). The pathophysiological metabolisms in MG patients vary depending on the thymic pathology. In thymomas, neoplastic epithelial cells contain muscle-specific antigens and have antigen-presenting properties besides abnormal expression; this includes defective expression of autoimmune regulator (AIRE) which is responsible for self-tolerance, absent thymic myoid cells, variable expression of striation all antigens epitope including titin and defective generation of Tregs. Thus, thymocytes selection and autoreactive T-cell export are remarkably influenced (8). During thymic atrophy, adipocytes in the thymus secrete a wide variety of cytokines and hormones that affect thymic and systemic immune function. Whether the development of adipocytes actively or passively induces immunosenescence during thymic involution remains unclear (9). In thymic hyperplasia, the thymic gland becomes a source of AChR antibodies, indicating the presence of germinal centers. The occurrence of lymphoid follicles with germinal centers transformed the role of thymus from T-cell maturation into a tertiary lymphoid organ with an intrathymic production of antibodies, compared to the peripheral generation in thymomas (10, 11). In case of post-thymectomy immune-mediated disorders, surgical resection removes a large fraction of thymus-resident autoreactive B cells. However, those that have migrated from the thymus could continue to cause disease because the autoantibody-producing B cell clones not only exist in the thymus but they can also populate compartments in the periphery. In addition, the distribution of pathogenic AChR autoantibody-producing cells in anatomic compartments other than the thymus, such as the bone marrow and lymph nodes, could be another reason for subsequent immune-mediated disorders (12).

There is increasing evidence that autoimmune diseases are commonly associated with MG, the most frequent being autoimmune thyroid disease (13–21). A systematic review by Mao revealed that the pooled frequency of comorbid autoimmune diseases was 13% (22). Similarly, in study by Shi, 92 of 796 Chinese MG patients had autoimmune comorbidities indicating that the prevalence of autoimmune diseases in MG was 11.6% (23). Central nervous system (CNS) involvement in MG, including neuromyelitis optica (NMO) (24), multiple sclerosis (MS) (25), autoimmune encephalitis (AE) (26), and inflammatory myopathy (IM) (27), has been reported. Although the prevalence of neurological autoimmune comorbidities in MG is low, there are multiple reports on this association. Here, we reviewed the studies on autoimmune diseases in MG to explore their clinical presentation and determine whether the presence of autoimmune diseases affects disease severity and treatment strategies for MG. Creating awareness of these comorbidities and performing the necessary testing may contribute to early comprehensive diagnosis and treatment.

## 2 Methods

We reviewed all the major immune-mediated co-existing autoimmune conditions associated with MG. A literature search was conducted using the PubMed, Embase and Web of Science Database without language restriction until January 2023. We used the following search terms: “myasthenia gravis” and “neuromyelitis optica” or “multiple sclerosis” or “autoimmune encephalitis” or “inflammatory myopathy” or “ autoimmune thyroid disorders” or “systemic lupus erythematosus” or “vitiligo”, “autoimmune disease”, “comorbidity”, “Sjogren’s syndrome”, “rheumatoid arthritis”, “autoimmune hemolytic anemia”, “autoimmune hepatitis”, “psoriasis”, “idiopathic thrombocytopenic purpura”, “pernicious anemia “and “scleroderma” respectively or in combination with “autoimmune diseases”, “autoimmune neurologic diseases”, “connective tissue disorder” and “complications”;

After duplicate studies were eliminated, all relevant articles were reviewed by two authors (Zhu Y and Hao YH) separately for considering the inclusion criteria, excluding review articles, comments/editorials, articles that were lack of relevant information. The reference lists of eligible articles were also reviewed in case of omission (Wang BQ). Controversies in data extraction between authors were confirmed by a third person (Zhu RX).

## 3 Results

### 3.1 Associated autoimmune disorders in AChR-MG

AChR Abs were found in 80% of MG patients. AChR-associated MG (AChR-MG) is strongly associated with thymus hyperplasia and thymoma, which is thought to play a crucial role in the disease pathogenesis. AChR-MG can be divided into early-onset MG (EOMG) and late-onset MG (LOMG), onset before or after age 50 years, respectively (4). The two groups differ with respect to thymic pathology, human leukocyte antigen (HLA) genotype, and autoimmune comorbidity. Titin and ryanodine receptor Abs always coincide with AChR-MG and are present in high frequency in thymoma. These can serve as biomarkers for severe MG. No association has been found between AChR Ab concentration and disease course, and concomitant MG and autoimmune diseases have been described in a number of case reports (1, 6, 7, 13). Most of these observations have focused on AChR-MG, while there are few reports on poly autoimmunity in MuSK-MG patients.

#### 3.1.1 Neurological autoimmune diseases

##### 3.1.1.1 Neuromyelitis optica

NMO is an aquaporin-4 (AQP4) Ab-mediated demyelinating disease that primarily affects the optic nerve and spinal cord. The coexistence of NMO and MG was recently documented. The association between them is unlikely a mere coincidence because the 2 diseases are rare, with MG prevalence at about 4-12 per million people (4) and AQP4-NMOSD prevalence at about 2.7 per million people (28). Both MG and NMO are B-cell-mediated humoral immunity disorders, and both AQP4 Ab and AChR Abs are IG1-complement activating Abs. AQP4 is expressed in the thymus gland and the abnormal thymus associated with MG is likely to generate specific autoantibodies such as anti-AQP4 Abs (29–31). B-cell activity and an imbalance between T-helper type 1 (Th1) and type 2 (Th2) cells may be essential for the development of MG and NMO. This could be attributed to the production of autoreactive T cells and the depletion of regulatory cells (32). Genetic predispositions, such as HLA subtypes and other environmental risk factors (vitamin D deficiency and evidence of EBV infection) could contribute to the co-occurrence of both diseases (24, 33, 34).

We identified 24 published articles in English with full texts or abstracts on concurrent MG and NMO cases in the English literature with available full text or abstracts, and the results are summarized in **Table 1**. A total of 70 cases were reported, including four case series. It is shown that that MG occurs before NMO in most patients (70%), and nearly 63% of patients have undergone thymectomy. Thymic pathology evaluation indicated that the majority had thymus hyperplasia while the others had thymoma or were normal. Thymectomy is a common therapy for MG and that NMOSD occurred with a latency of up to several years; therefore, the occurrence of NMO after thymectomy could be a coincidence. Leite et al. first summarized the details for a cohort of 16 patients with coexistence of MG and NMO and AChR and AQP4 Ab (29). Only two patients with no history of thymectomy were initially diagnosed with NMO and later developed MG. The patients presented with early-onset generalized AChR-MG, and a high proportion achieved remission without crisis. AQP4-Abs are detectable years before the onset of MG without NMO symptoms (29, 35). MG at NMO presentation rarely relapsed and AChR and AQP4 Ab levels presented opposite tendency (29). Jarius et al. conducted a retrospective study on 26 patients mainly of Caucasian origin (36). The age of MG and NMO onset among Caucasian patients was identical to that usually observed in EOMG. In addition, the median latency between thymectomy and the onset of NMO was shorter in the Caucasian group. More recently, Iyer et al. summarized 58 reported cases of concurrent NMO and MG and concluded that NMO occurred after thymectomy (19). The levels of anticardiolipin and anti-double-stranded DNA Abs and ANA titers of anti-nuclear Abs (ANAs) were higher in patients with MG who underwent thymectomy than that in those who did not.

**Table 1:** Characteristics of patients diagnosed with the coexistence of NMO and MG.

Author	Number	Sex	Age of MG	MG type	Age of NMOSD	NMOSD Presentation	MG Antibody	NMOSD Antibody	Thymectomy
O’Riordan 1995	1	F	Previous positive history	NA	41	ON	AChR Ab (+)	NA	NA
Isbister 2003	1	F	28	G	36	ON, TM	AChR Ab (+)	NA	Yes (hyperplasia)
Antoine 2004	1	M	49	G	49 (4 months after MG)	TM	NA	NA	Yes (thymoma)
Kister 2006	4	4F	38,36,17,27	2O, 2G	39,41,19,38	3 ON, 1 ON, TM	4 AChR Ab (+)	1 NMO-Ig, 1 NMO-IgG, anti-GAD, ANA, 1 ANA, 1 NMO-IgG	Yes (hyperplasia)
Furukawa 2006	2	2F	23,NA	2G	48,63	2TM, ON	2 AChR Ab (+)	No	Yes (hyperplasia), No
Gotkine 2006	1	F	10	G	26	TM	AChR Ab (+)	NA	Yes
Ikeda 2007	1	NA	29	G	53	ON, TM	–	NA	Yes (hyperplasia)
Nakamura 2007	1	F	28	NA	38	ON	AChR Ab (+)	+	Yes
Bichuetti 2008	1	F	27	G	31	TMO	NA	NA	Yes (hyperplasia)
Kay 2008	1	F	44	O	40, 49	ON	AChR Ab (+)	AQP4-IgG	No
Uzawa 2009	2	2F	20,18	2G	41,27	2 ON	2 AChR Ab (+)	AQP4-IgG	Yes (1hyperplasia, 1NA)
Kohsaka 2010	1	F	Previous positive history	NA	60	TM	NA	AQP4-IgG	Yes (hyperplasia)
Etemadifar 2011	1	F	40	G	33	ON	AChR Ab (+)	AQP4-IgG	No
Etemadifar 2011	1	F	42	G	33	ON, TM	AChR Ab (+)	AQP4-IgG	No
Vaknin-Dembinski 2011	15	10F,5M	NA	NA	NA	OM, TM	11 AChR Ab (+)	7 AQP4-IgG	9 Yes (7 thymoma, 2 hyperplasia), 6 No
Leite 2012	16	15F,1M	12-47(26.5)	13G, 3O	23-67(39.5)	8 ON, 8 TM	16 AChR Ab (+)	AQP4-IgG	11 Yes (8 hyperplasia, 3 normal histology), 5 No
Jarius 2012	10	10F	11-33	NA	20-67	5 TM, 5 ON	9 AChR Ab (+), 1 NA	AQP4-IgG	6 Yes (3hyperplasia, 2normal, 1 thymitis), 4 No
Ogaki 2012	1	F	30	G	43	ON	AChR Ab (+)	AQP4-IgG	Yes
Hironishi 2012	1	F	23	G	30	ON	NA	–	Yes (hyperplasia)
Spillane 2013	1	F	23	G	31	TM, APS	AChR Ab (+)	AQP4-IgG	Yes (normal histology)
Ikeguchi 2014	3	2F, 1M	25,41,47 (37.7)	3G	49,53,70 (57.3)	2TM, 1ON	3 AChR Ab (+)	2 AQP4-IgG,1 NA	2 Yes (1 thymoma, 1 normal), 1 No
Balarabe 2015	1	F	8	G	14	ON	AChR Ab (+)	AQP4-IgG	No (thymic enlargement)
Castro-Suarez 2020	2	2F	20, 22	1 NA, 1O	49,23	1 ON, TM 2 TM	2 AChR Ab (+)	1 NA, 1 AQP4-IgG	2 Yes (hyperplasia)
Bates 2020	1	F	39	NA	54	TM	AChR Ab (+)	AQP4 and anti-MOG antibodies	Yes

O, Ocular; G, Generalized; ON, Optic Neuritis; TM, Transverse Myelitis; NA, Not Available.

From these cases, we concluded that the clinical presentation of MG preceded the onset of NMO by up to a decade in the majority of the cases (37–42), especially in EOMG patients. Thymic hyperplasia is more common in patients with MG and NMO. Notably, MG combined with NMO presents a benign course with long-term remission without immunosuppressive drug administration. MG remained in remission in most patients and was not influenced by the course of NMO in this case series. We speculate that this interesting phenomenon occurs because thymectomy is an effective treatment for MG; it keeps the condition relatively stable. However, NMOSD is often severe and can lead to disabilities. The opposite trends in AChR and AQP4-Ab levels could also be one of the reasons. Therefore, NMO should be considered and serum AQP4 Ab levels should be tested in MG patients with clinical signs or symptoms indicative of NMO; these include spinal cord and optic neuropathy, and brainstem symptoms such as intractable vomiting, narcolepsy or hiccoughs. Understanding the characteristics of these comorbidities is important, and further research is needed to address the common immunobiological mechanisms underlying NMO and MG.

##### 3.1.1.2 Multiple sclerosis

MS is a chronic autoimmune demyelinating disease of the CNS characterized by peripheral immune cells, such as T cell/Th17-type and macrophage infiltration, demyelination, axonal injury, and neuronal damage. The incidence of MS in the adult population is estimated to be 35 per million people in the United States (43). The loss of self-tolerance plays an essential role in the pathogenesis of both disorders, leading to the development of autoreactive lymphocytes and suppression of effector CD4+ T cells by Tregs, which mediates autoimmune responses and maintains self-tolerance (44). Autoantibodies against the neuromuscular junction protein. AChR cause MG, in which the autoimmune response is initiated by the activation of T lymphocytes. In addition, B cells and autoantibodies have a major influence on MS pathogenesis (45). Therefore, both humoral and cell-mediated immunity play a role in the pathogenesis of co-occurrence of these two diseases. In addition, the main LOMG HLA risk allele DRB1*1501 is associated with MS risk (20, 46).

To date, we summarized 29 cases of MG co-occurring with MS and the results are summarized in **Table 2**. An epidemiological study in 1989 from Finland found 2 patients in a population of 1.5 million had both diseases (47). Gotkin et al. reported that 2.3% of MG patients have MS, supporting a nonrandom association (48). Lorenzoni et al. first reviewed the previous cases and concluded that patients with both diseases were predominantly female, often manifested a mild clinical course, and rarely underwent MG crisis (49). Sylvester et al. reviewed 19 cases of MG co-occurring with relapsing-remitting MS (RRMS) in 2013 (50). The diagnosis of MS preceded MG in ten (53%) cases, and MG preceded MS in three (16%) cases. MS may develop prior to or following MG. Subsequently, Dehbashi et al. in 2019 confirmed these findings in 29 cases of co-occurrence of both diseases (25). The occurrence of MG could exacerbate the course of MS, whereas MG can rarely be influenced by fluctuations because of MS. Furthermore, a Norwegian study reported that LOMG has a higher risk of coexisting with MS than EOMG (46). Nevertheless, we found that MS were prone to coexist with EOMG (86%) than LOMG. In addition, ANAs were found to be abnormal in patients with both diseases. The co-occurrence of MS and AChR-MG was more frequent than that of MS and MuSK-MG (50, 51). Similar immunogenetic backgrounds are more likely to lead to susceptibility to the coexistence of the two disorders, but unknown genetic factors and different provocative factors lead to two different diseases (47). Additionally, recent studies have shown that MS and MG share HLA loci, which may explain the co-occurrence of these diseases.

**Table 2:** Characteristics of patients diagnosed with the coexistence of MS and MG.

Author	Number	Sex	Age of MG	Age of MS	MS Presentation	MG Antibody	MS Antibody	Thymectomy
Margolis 1945	1	F	25	41	NA	NA		No
Patten 1972	1	F	26	27	NA	NA		No
Aita 1974	4	3F, 1M	22, 17, 22, 59	24, 45, 30, 48	NA	NA		3 Yes (hyperplasia), 1 No
Achari 1976	1	F	32	23	NA	NA		No
Lo 1983	1	F	25	26	NA	AChR Ab (+)		Yes (hyperplasia)
Shakir 1983	1	F	18	25	NA	AChR Ab (+)		Yes (hyperplasia)
Somer 1989	2	F	21, 36	21, 32	Diplopia, weakness, clumsiness and numbness of upper extremity	AChR Ab (+)	OB	2 Yes (hyperplasia)
Isbister 2003	8	7F, 1M	28,35,20,30,28,43,41,27	36,28,26,26,20,34,37,34	Optic neuritis, Weakness,balance, Parasthesia, Bell’s palsy (6 RRMS)	4 AChR Ab (+), 3 (-), 1 NA		3 Yes (hyperplasia), 5 No
Basiri 2009	6	5F, 1M	16, 40, 52, 38, 21, NA	23, 31,51, 38, 19, NA	Lower limb paresthesia, Optic neuritis, Facial palsy (6 RRMS)	AChR Ab (-)		5 Yes (hyperplasia), 1 NA
Gharagozli 2011	1	F	44	41	RRMS	AChR Ab (-)		No thymic enlargement
Sylvester 2013	1	M	50	48	Lethargy, intermittent diplopia, phonophobia, episodic slurred speech, unsteadiness of gait, short-term memory loss, and impaired concentration (RRMS)	MuSK Ab	OB	No thymic mass
Alanazy 2018	1	M	18	18	Bilateral lower limb numbness	seronegative		No
Dehbashi 2019	1	M	54	35	Internuclear ophthalmoplegia (RRMS)	AChR Ab (+)		Yes (thymic normal)

NA, Not Available.

Female patients have a predisposition for the co-occurrence of RRMS and EOMG based on current cases (15 RRMS out of 29 cases); however, there are few reports on other types of MS and further clinical observations are needed. The clinical diagnosis of MS can be made before or after the occurrence of MG. The presentation of these two diseases is less severe and they are not prone to MG crises. Patients with concomitant MS and MG usually present with mild symptoms of MS. Sixteen patients among the 29 cases underwent thymectomy; 15 had thymic hyperplasia and one was thymically normal. Therefore, thymic hyperplasia could be more common in patients with MG and MS.

##### 3.1.1.3 Autoimmune encephalitis

Autoimmune encephalitis (AE) is characterized by cognitive decline, seizures, loss of consciousness, and mental confusion due to Abs against neuronal cell surface and synaptic proteins. The thymus is important for the selection of T cells with self-tolerance and is regarded as an initiator for AE. Besides MG, approximately 10-15% of thymomas present with as limbic encephalitis (52–56).

Up to now, fifteen cases of concurrent MG and AE were identified and the results of the literature review are summarized in **Table 3**. In 2009, Monstad et al. first reported a patient who developed AE due to collapsing response mediator protein 5 (CRMP5) Abs 12 months after the diagnosis of MG (57). Anti-CRMP5 Ab is usually associated with small cell lung cancer, paraneoplastic neurological syndrome and thymoma, manifesting with different disorders of the central and peripheral nervous systems. In 2015, Li et al. described a patient with thymomatous MG who developed AMPAR encephalitis (58). The patient, who first presented with behavioral and mental dysfunction, was diagnosed with AMPAR encephalitis and received steroid treatment. However, 6 months later, the patient developed MG based on dysarthria and dysphagia and underwent thymectomy. At the last follow-up, she was neurologically asymptomatic except for mild cognitive impairment. Besides AMPAR-Ab and AChR-Ab, the patient was positive for titin-Ab. Moreover, other patients with co-occurrence of MG and AE have been reported to be positive for titin-Abs. In 2019, Luo et al. described a female MG patient with AMPAR encephalitis onset after a complete thymectomy. The patient was diagnosed with type B2 thymoma-associated AChR MG and underwent thymectomy. Two years later, she presented with memory loss, behavioral changes, and AMPAR positivity and was diagnosed with anti-AMPAR encephalitis. Subsequently, she underwent immunotherapy, and her symptoms gradual improved (59). Additionally, Hor et al. demonstrated that an elderly man who presented with nephrotic syndrome was diagnosed with thymoma-associated AChR MG and LGI1-encephalitis after thymectomy in 2018 (26). The main symptoms were memory deterioration, mild confusion, and personality changes. The patient underwent thymectomy and immunotherapy, and the symptoms gradually improved. In 2022, a case complicated with LGI1 encephalitis and thymoma-associated AChR MG was reported by Satake et al (60). The patient first presented with memory impairment and had bilateral ptosis. Serum Ab tests were positive for LGI1 Ab, titin Ab, and AChR Ab. Therefore, she underwent thymectomy and immunosuppressive therapy. The patient showed marked improvement in symptoms with immediate amelioration of motor weakness.

**Table 3:** Characteristics of patients diagnosed with the coexistence of AE and MG.

Author	Number	Sex	Age of MG	MG symptom	Age of AE	AE presentation	MG Antibody	AE Antibody	AE treatment	Thymectomy
Kodama 1991	1	F	34	dysphagia, lethargy and diplopia	34	Memory deficit, psychiatric abnormality, language dysfuncation	NA	NA	NA	Yes
Evoli 1999	1	M	33	fluctuating ptosis and weakness of upper limb proximal muscles	32	Seizures, memory deficit, psychiatric abnormality, language dysfuncation, neuromyotonia	AChR Ab	NA	chemotherapy (cyclophosphamide, doxorubicin, vincristine and metilprednisolone)	Yes
Buckley 2001	1	F	47	NA	58	Memory deficit, psychiatric abnormality	AChR Ab	VGKC	loxapine, immunosuppression	Yes
Vernino 2002	1	M	34	dysarthria and dysphagia	34	Seizures, memory deficit, psychiatric abnormality, language dysfuncation	AChR Ab	Hu	phenytoin and quetiapine	Yes
Khella 2007	1	F	41	diplopia, dysphagia, and neck weakness	41	Memory deficit, psychiatric abnormality, cerebellar symptom	AChR Ab	unknown antibody to neuronal cytoplasm	steroid and IV gamma globulin	Yes
Monstad 2009	1	F	48	right ptosis	50	Memory loss; behavioral changes	AChR Ab	CRMP5	steroid and azathioprine	Yes
Hammoud 2009	1	F	43	fatigable weakness, dysarthria, diplopia, and ptosis.	47	Seizure and confusion	AChR Ab	VGKC	steroid And IVIg	Yes
Storey 2010	1	M	67	NA	73	Headache and fever over 2 weeks and a transitory loss of consciousness.	AChR Ab	NA	immunotherapy and thymectomy	Yes(thymus atrophy)
Miyazaki 2012	1	M	46	ptosis and diplopia	50	Seizures, memory deficit, psychiatric abnormality, language dysfuncation	AChR Ab	VGKC	Steroid	Yes (thymoma)
Shaulov 2012	1	F	84	dysphagia, and extreme tiredness	66	Seizures, memory deficit, psychiatric abnormality	AChR Ab	NA	Anti-epileptic treatment	Yes
Miyazaki 2013	1	M	46	ptosis and diplopia	50	Visual hallucinations; generalized seizures	AChR Ab	VGKC	Steroid	Yes (thymoma)
Li 2015	1	F	48	dysarthria and dysphagia	47	Progressive apathy; aggressive behavior	AChR Ab; titin Ab	AMPAR	steroid and azathioprine	Yes (thymoma)
Hor 2018	1	M	69	left ptosis, dysphagia, dysarthria	70	Memory deterioration; personality changes	AChR Ab	LGI1	steroid And IVIg	Yes (thymoma)
Luo 2019	1	F	48	right ptosis	50	Memory loss; behavioral changes	AChR Ab	AMPAR	steroid and azathioprine	Yes (thymoma)
Satake 2022	1	F	77	bilateral ptosis, and left dominant facial weakness	77	Memory impairment	AChR Ab; titin Ab	LGI1	Steroid	Yes (thymoma)

NA, Not Available.

In these cases, the onset of MG was usually prior to AE (53%) except that a few cases of patients developing MG concurrently with AE or after the onset of AE. The VGKC (LGI1 and CASPR2) Ab is more commonly associated with AE than other Abs. Thymoma is a common cause of paraneoplastic disease and associated with an increased risk of AE. Titin Ab have a high positive predictive value for thymoma and are found to be associated with AE. Timely thymectomy followed by immunotherapy was widely believed to be an effective treatment for two co-occurring diseases. Clinicians should be aware that patients with MG who present with atypical clinical characteristics such as mental dysfunction or memory impairment may have AE.

##### 3.1.1.4 Inflammatory myopathy

Idiopathic inflammatory myopathies (IMs) are a heterogeneous group of muscle disorders including polymyositis (PM), dermatomyositis (DM), inclusion body myositis, immune-mediated necrotizing myopathy, and overlap myositis, with an annual incidence rate of approximately 19 per million people (61). MG and IMs are both acquired diseases caused by autoimmune responses against neuromuscular junctions and muscle tissues, respectively. Although both 2 diseases are rare, there have been cases of MG and IM co-occurrence, and it is increasingly being recognized that the comorbidity of MG and IMs could be related to thymoma as a paraneoplastic phenomenon (62–68).

We identified 69 patients with myositis. The clinical, laboratory, and electrophysiological features of the patients are summarized in **Table 4**. The Johns Hopkins Neurology Center Group first reported six patients with co-occurring MG and IM (four PM and two DM), including two with thymic hyperplasia in 2014 (63). Similarly, Santos et al. reported four patients with MG and IM (two PM and two DM): two with thymic pathology and two without in 2017 (69). More recently, in a Italy cohort, Garibaldi et al. identified 13 PM with co-occurring MG, ten of them had thymoma (70). The presence of thymomas in patients with co-occurring MG and IM suggests that thymoma-related immunopathogenic mechanisms, including dysregulation of the immune checkpoint pathway, could contribute to the coexistence of these diseases (71). A large cohort study found that anti-AChR was detected in all patients with MG and IM associated with anti-titin and anti-RyR1 antibodies in patients with thymoma; however, muscle-specific antibodies Abs (MSAs) and myositis-associated autoantibodies were not detected (70). In most patients, MG-IM patients occurred simultaneously and it commonly co-existed with LOMG rather than with EOMG. In addition, immune checkpoint inhibitors, such as programmed cell death 1 or cytotoxic T-lymphocyte-associated protein 4 could induce these diseases by regulating T cell activation instead of thymoma (70).

**Table 4:** Characteristics of patients diagnosed with the coexistence of IM and MG.

Studies	Number	Age and Sex	Initial Presentation	Myositis type	Thymoma	Treatment	anti-AChR	MSAs	anti-titin Ab
Pascuzzi 1986	1	82F	MG	Granulomatous inflammatory myopathy	Unknown	Glucocorticoids	1+		
Hassel 1992	1	37M	MG and PM	PM	Thymoma of cortical type	Thymectomy, glucocorticoids, CsA, PLEX	1+		
Ko 1995	1	25F	MG and PM	PM	Thymoma of cortical type	Thymectomy, glucocorticoids, pyridostigmine, and AZA	1+		
Hausmanovwa 1995	1	58F	MG	DM	None mentioned	Glucocorticoids and pyridostigmine	NA	PM-Scl	
Kobayashi 1997	1	14F	MG	PM	Thymoma	Ambenonium, thymectomy, and glucocorticoids	NA		
Ikeda 1998	1	36F	MG	Eosinophilic infiltration and myopathy	Benign thymoma	Thymectomy, glucocorticoids, and PLEX	1+		
Raschilas 1999	1	66F	NA	PM	Malignant thymoma	Glucocorticoids and pyridostigmine	NA		
Kornizky 2000	1	69F	MG	PM	None found	Glucocorticoids and MTX	1+	ANA, anti-U1RNP	
Otton 2000	1	71M	MG	PM	Thymic mass on CT	Glucocorticoids and AZA	1+		
Van de Warrenburg 2002	1	28F	MG and DM	DM	None found	Glucocorticoids and pyridostigmine	1+	AMA	
Diaco 2004	1	47F	MG	PM	Thymic hyperplasia	Pyridostigmine and thymectomy, glucocorticoids, MTX, and CsA	1+	Jo-l	
Tanahashi 2004	1	62M	MG	Giant cell myocarditis and myositis	Invasive thymoma	Extended thymectomy and radiotherapy glucocorticoids, and tacrolimus	1+		
Shichijo 2005	1	57M	MG and DM	DM	None found	Glucocorticoids	1+	ANA, anti-DNA	
Avni 2006	1	66M	MG and PM	PM	Thymoma	Glucocorticoids and IVIG	1+		
Yoshidome 2007	1	62F	MG and PM	PM	None found	Glucocorticoids and IVIG	1+		
Suzuki 2009	8	45M, 43F, 44F, 68F, 66F, 48F, 66F, and 62F	3MG, 2PM, 3 MG and PM	Myocarditis was found in 3 patients and myositis in 6 (1 patient had both).	Four had invasive thymoma included types AB, B1, and B3	Eight received glucocorticoids; 5, PLEX; 5, Tac; 2, IVIG; and 2, CsA	8+		5+
Hill 2011	1	67F	MG and DM	DM	None found	Glucocorticoids and IVIG	1+		
Sasaki 2012	1	58M	Giant cell myocarditis and myositis	Giant cell myocarditis and myositis	Stage IVa thymoma	Carboplatin and paclitaxe, Passed away from respiratory failure	1+		
Seton 2013	1	46F	NA	PM	Type B1 thymoma	Glucocorticoids, pyridostigmine, and thyomectomy	NA		
Kon 2013	1	80F	MG	Giant cell polymyositis and myocarditis	Thymoma B1	Passed away from extensive myocarditis and respiratory failure	1+		
Paik 2014	6	75F, 44M, 54M, 38F, 61M, 24F	Co-existence	2 DM and 4 PM	Two thymic hyperplasia by CT chest	Pyridostigmine, IVIG, glucocorticoids, thymectomy, and MMF	4+	1 Anti-P155/140, 1 Anti-PM/Scl	
Lin 2014	1	40F	MG and Giant cell myocarditis	Giant cell myositis	Thymoma B3	Glucocorticoids, neostigmine, thymectomy, and radiation	1+	ANA1:100, pANCA	
Santos 2017	4	32M, 33F, 38M, and 69M	3 MG, 1 DM	2 PM and 2 DM	One with type C thymoma (thymic carcinoma) and one with thymolipoma	MMF, MTX, AZA, and glucocorticoids	3+	Anti-gastric parietal cells Ab, ANA 1:160, Anti-Pl7 Ab, ANCA positive 1/640	
Huang 2019	7	29F, 44F, 52F, 66F, 25M, 49F, 41F	4MG, 2 MG and DM, 1MG and PM	2 DM and 5 PM	Four thymoma included types AB and B1	Thymectomy, IVIG, Pred, chemo and MTX	6+	PL-7	
Uchio 2019	10	6F,4M,Mean age=60	9MG, 1PM	10PM	Seven thymoma included types B2 and B3	Thymectomy, IVMP, PSL, TCR, CyA, PB, IVIg, IAPP, MTX	9+		6+/8
Garibaldi 2020	13	7F,6M,Mean age=51	2MG, 10 MG and PM, 1PM	13PM	Ten thymoma included types AB and B2	Ten Thymectomy, PD, CYA, AZA, MTX, IVIG, PEX	13+		All tested patients with thymoma presented also anti-titin and -RyR1 Ab
Villalb 2020	1	66	MG and PM	PM	None mentioned	IVIG, prednisone, Pyridostigmine and azathioprine	1+		

PM, polymyositis; DM, dermatomyositis; NA, Not Available.

More than 60% of patients with co-occurring MG-IM overlap had an underlying thymoma, indicating that thymus pathology plays an important role in the origin of the disease association. The majority of the co-occurrence of MG-IM is PM with thymoma, based on the current data (44 out of 69 cases). IM occurs simultaneously with MG (46%), and it commonly coexists with LOMG (67%). Most patients were positive for AChR Abs associated with titin Abs, RyR1 Abs associated with thymomas, and negative for MSAs.

#### 3.1.2 Systemic autoimmune diseases

##### 3.1.2.1 Thyroid disease

Autoimmune thyroid disease (AITD) leads to autoimmunity against thyroid antigens and includes three major diseases: Graves’ disease (GD)-triggered hyperthyroidism, hypothyroidism due to Hashimoto’s thyroiditis (HT), and euthyroid patients with positive antithyroid Abs (72). Many studies indicated that AITD most commonly co-occurs with MG and affect its clinical course (18). HLA-DR3 and B8 antigens may cause a predisposition to the development of other autoimmune diseases in EOMG and are often seen in GD patients (73, 74).

Increasing evidence showed that the prevalence and risk of thyroid disorders are higher in MG patients than in the general population. Chou et al. revealed that the prevalence of thyroid diseases in the MG patients was 18.4%, which was nearly 3.9-fold greater than that in the control group (75). Another meta-analysis reported the pooled estimate of thyroid autoimmunity incidence in MG patients was 10.1% (17). A retrospective study by Sehgal et al. indicated that MG patients with positive thyroid Abs had more AChR Abs and more abnormalities in T cells than the control group (76). In addition, AITD varied between the MG subgroups. Patients with EOMG had a much higher frequency of AITD than those with LOMG (76). AITD occurred in up to 10% of EOMG patients and manifested as thyroid deficiency or excess (22). Meanwhile, Kubiszewska et al. reported that MG patients with AITD required immunosuppressive treatment less frequently and therefore had a milder clinical course than patients with MG alone (18). The most commonly associated condition in patients with MG and AITD is ocular MG at onset (23). Especially, previously published data indicate that MG associated with AITD has mild clinical course with more common ocular involvement (18, 77). Ocular symptoms occur in approximately 15% of patients with MG (78). Ocular MG has a special link with thyroid disorders, which may be interpreted as immunological cross-reactivity against autoantigens or epitopes co-expressed by the eye muscles and thyroid (79–81). In addition, patients with MG and autoimmune disease were less susceptible to myasthenia crisis (MC) and were prone to have a mild clinical presentation (79, 82).

AITD is the most common autoimmune comorbidity of MG with an incidence of 10.1%, especially in EOMG. The most commonly associated condition in MG patients with AITD is ocular MG at onset, while the generalized type is rare. Furthermore, patients with MG-AITD are less susceptible to MC and tend to have mild clinical presentation.

##### 3.1.2.2 Systemic lupus erythematosus

SLE is a multisystem autoimmune disease with protean manifestations caused by the presence of various autoantibodies that cause chronic inflammation. The prevalence of SLE in MG is 2.2-8.3%, and the prevalence of MG in SLE is 1.3%, which is remarkably higher than the prevalence of MG in the general population (0.02%) (83–85). It has recently been highlighted that the α chemokine subfamily (CXC) is involved in the pathogenesis of both disorders. CXCL13 is a chemokine that activates B and T lymphocytes, which further contribute to the pathogenesis of SLE and MG (86, 87). Evidence which exists to also support that a common genetic background (HLA B8 and HLA-DR3) may explain the coexistence of MG and SLE (88, 89).

MG preceded SLE in more than twice as many cases (62%); both conditions rarely presented simultaneously (85, 86). Patients with MG and SLE were younger, showed a higher prevalence of AChR-Ab, received more immunosuppressants, underwent thymectomy more frequently, and presented a higher rate of remission than those with MG only (90). As autoimmune disease were more prevalent in women than in men, most patients with MG-SLE were women. Jallouli et al. showed that the clinical course of SLE was milder with less frequent cutaneous and renal manifestations in MG patients than those with SLE alone (85). In most cases, MG occurs before SLE, and thymectomy is a precipitating factor for the development of SLE due to the loss of central tolerance and overproduction of antibodies. Polyarthritis is the most common manifestation of SLE after thymectomy (91). Iwadate et al. reported a 49-year-old woman with pure red cell aplasia, SLE, and idiopathic portal hypertension 9 years after thymectomy (92). Another possible reason related to the MG-SLE overlap is hydroxychloroquine, which has a direct effect on neuromuscular junctions producing myasthenia-like syndrome and even triggering MG relapses (85). Hence, when symptoms persist despite hydroxychloroquine withdrawal in SLE patients, MG should be ruled out.

MG preceded SLE in more than twice as many patients, with the majority being female. SLE more frequently co-occurs with EOMG than with LOMG. SLE-MG patients showed a higher prevalence of anti-AChR Abs, received more immunosuppressants, more frequently underwent thymectomy, and had a milder clinical course of SLE with less frequent cutaneous and renal manifestations than SLE patients.

##### 3.1.2.3 Vitiligo

Vitiligo is a pigmentary disorder that is characterized by white patches in the skin due to dysfunction of melanocytes in the skin or hair. MG coexisting with vitiligo was first recognized by Durance in 1971 (93). However, to date, there are few case reports of MG co-occurring with vitiligo. The frequency of vitiligo in MG patients was 1.3-fold higher than that in the general population. Although results vary, the prevalence of MG in vitiligo patients and vitiligo in MG patients is 0.2% and 1.7%, respectively (94–97).

##### 3.1.2.4 Sjogren’s syndrome

SS is an autoimmune disease characterized by xerophthalmia and xerostomia caused by lymphocytic infiltration of the exocrine glands (salivary and lacrimal glands). SS is a B-cell mediated autoimmune disease with anti-SSA (Ro), anti-SSB, and ANA Abs. The frequency of neurological involvement, such as peripheral neuropathy, aseptic meningitis, NMO, and MS in SS is up to 20% (98–100). However, the coexistence of MG and SS has rarely been reported. Li et al. reviewed 17 patients with both diseases and revealed that MG may develop before or after SS (100). The coexistence of both diseases predominantly affected female and EOMG patients. Co-morbidity with MG does not seem to adversely affect the course of SS. Berrih-Aknin et al. found that HLA-DR3 was associated with an increased risk of coexistence of MG and SS (101).

##### 3.1.2.5 Pernicious anemia

Pernicious anemia is an autoimmune disease caused by atrophic gastritis; it produces autoantibodies against gastric parietal cells or intrinsic factors. This impairs the absorption of vitamin B12 (102). Neurological disorders usually present with subacute combined lateral degeneration (103). However, there are few case reports on the coexistence of MG and pernicious anemia. Simpson et al. first reported 9 cases of pernicious anemia in 491 patients with MG, with an incidence of 1.83%, and concluded that the incidence of pernicious anemia in patients with MG was higher than that in the normal population (104). Chang et al. reported the case of a 73-year-old woman with a positive AChR Ab and without thymoma who developed MG 5 months after the onset of pernicious anemia. Her MG and pernicious anemia symptoms markedly improved after treatment with pyridostigmine, prednisolone, and hydroxocobalamine (105). When MG presents with anemia or posterior column symptoms, pernicious anemia must be ruled out because of the beneficial treatment effects for both diseases.

##### 3.1.2.6 Other autoimmune diseases

MG also coexists with other autoimmune diseases such as rheumatoid arthritis, autoimmune hemolytic anemia, autoimmune hepatitis, psoriasis, idiopathic thrombocytopenic purpura, and scleroderma; however, these are rarely reported. Additionally, autoantibodies such as ANA and anti-ds-DNA can be found in MG without clinical indication of related SS and may be marker for the late development of autoimmune disease in some patients. Patients with MG are recommended for testing of thyroid-related Ab.

### 3.2 Associated autoimmune diseases in MuSK-MG

MuSK-MG accounts for 1-10% and 10-70% of MG cases and all AChR negative MG cases, respectively. Bulbar weakness is a common symptom in patients with MuSK-positive MG. Nevertheless, limb and ocular muscles are usually not involved (106). MuSK Abs that belong to the IgG4 subclass are directly pathogenic and do not activate the complement pathway. They show great heterogeneity in clinical presentation and pathogenesis. Shi et al. proposed that the proportion of MuSK positivity was higher in MG patients without autoimmune diseases than in those with them (23). Thus far, autoimmune diseases associated with MuSK-MG have rarely been reported. MuSK-positive MG was rarely accompanied by autoimmune diseases because thymic abnormalities were not involved in the pathogenesis of MuSK-MG. HLA-DQ5DR14 and DR16 are associated with MUSK-associated MG, which is different from other MG subgroups (4). Sylvester et al. presented the only case of MuSK-associated MG co-occurring with RRMS (50). Therefore, given the limitation of small numbers of MuSK-MG patients, additional cases should be identified in future studies.

### 3.3 Associated autoimmune diseases in LRP4-MG

The low-density LRP4 Abs were detected in 1-2% and 2-27% of total MG cases and both AChR and MuSK negative MG cases, respectively. LRP4 Abs that belong to the complement-binding IgG1 subclass inhibited AChR clustering in the membrane by blocking the agrin-LRP4 interaction. Most of these patients present with ocular or mild generalized MG. The thymus was found to be normal in most patients with LRP4-associated MG. Co-occurrence of autoimmune diseases in LRP4-MG patients has rarely been reported, whereas LRP4 Abs were detected in 23.4% of patients with amyotrophic lateral sclerosis (107, 108).

## 4 Outlook

Based on the above findings, we found that AChR-MG had a higher frequency of comorbid autoimmune diseases than MuSK-MG and LRP4-MG, which was consistent with the results of Shi’s study (23). Autoimmune diseases was more common in EOMG than in LOMG patients. Additionally, the frequency of concomitant autoimmune diseases in MG patients is approximately 13%. Thyroid disorders were the most common system autoimmune diseases together with SLE and vitiligo. Other autoimmune diseases such as SS, rheumatoid arthritis, autoimmune hemolytic anemia, pernicious anemia, autoimmune hepatitis, psoriasis, idiopathic thrombocytopenic purpura, and scleroderma were found to coexist with MG. Consistent with previous reports, a unique link between ocular MG and AITD was found. Moreover, our study provides evidence that vitiligo was a more common comorbidity that occurred with autoimmune diseases than did previously reported reviews and this should be taken into consideration in clinical practice. The proportion of MGFA I type at onset in MG coexisting with autoimmune diseases was higher than those in MG without autoimmune diseases, indicating that coexisting conditions have a mild course. Furthermore, autoimmune diseases appear to develop more often in women than in men.

We first reviewed published articles on concurrent MG and neurological autoimmune diseases. Of note, MG can coexist with neurological autoimmune diseases such as NMO, MS, AE, and IM. Previous reports indicate that 2% of patients with NMO have clinical MG (34). Moreover, Gotkine et al. reported five of 214 reviewed patients with MG (2.3%) who had CNS demyelinating disease (48). Our review reports that the probability of co-occurrence of MG and autoimmune diseases is much higher than what was expected by chance. It has been suggested that NMO, IM, and AE often occur after the diagnosis of MG, and MS may develop before or after the onset of MG. Additionally, thymoma-associated AChR MG was linked with the increased risk of AE and IM while NMO and MS were mainly related to thymic hyperplasia. Therefore, timely immunotherapy after thymectomy may be an effective treatment for preventing the co-existence of two diseases. Female patients with MG have a higher risk of coexistence of MG and neurological autoimmune diseases than male patients with MG. IM and RRMS are prone to coexist with LOMG, whereas EOMG patients are susceptible to NMO. Importantly, patients with MG combined with neurological autoimmune diseases are less likely to experience MC and have a milder clinical course than those with only MG. The coexistence of AD did not influence the clinical progression and prognosis of MG. MG at NMO presentation rarely relapsed and AChR and AQP4 Ab levels presented opposite tendency. However, the course of neurological autoimmune diseases is often severe. Additionally, we identified that MG patients with thymoma were susceptible to the development of AE and IM. Striational proteins antibodies have been found in patients with MG and IM such as titin, ryanodine receptor, and muscular voltage-gated potassium channels (Kv1.4) (109, 110). It has been suggested that thymoma MG is closely associated with the coexistence of IM and AE.

**Figure 1:**
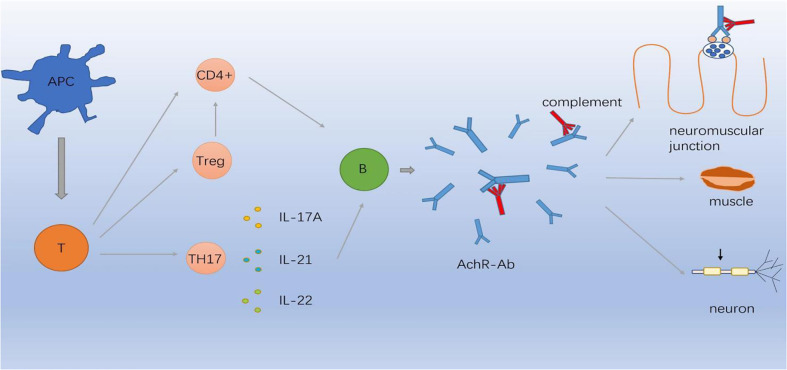
Immunopathogenesis involving different targets in MG. APC cells differentiate in the periphery to T cells capable of producing CD4-positive (CD4+) T cells, Treg cells and Th17 cells which secret IL-17A, IL-21, IL-22. Abnormal inflammatory environment induces complement through B cells produce AChR-Ab which binds to AChR, causing neuromuscular junction conduction disorder.

It is noteworthy that autoimmune diseases were mostly complicated by thymoma, revealing an immunological link between the CNS and muscles. Furthermore, thymic abnormality may play a role through immune dysregulation or genetic predisposition and may share similarities with autoimmune diseases in terms of immunological, environmental, epigenetic, and genetic factors. Most autoimmune diseases are thought to result from the loss of self-tolerance. Tregs exert essential role in maintaining self-tolerance and inhibiting the activity of CD4+ T lymphocytes, finally halting the autoimmune process. It is well documented that functional deficits in Tregs may contribute to the pathogenesis of multiple autoimmune diseases including MG, MS, and NMO. B cells coinciding with Treg dysfunction were found to be hyperreactive in most autoimmune diseases and generated excessive autoantibodies. Specific autoantibodies and autoreactive T cells appear to share characteristics of MG and autoimmune diseases. However, the potential pathogenesis of the coexistence autoimmune disease remains to be further explored. Recently, complement dysfunction is now recognized to be involved in core of pathogenesis of autoimmune diseases. Thereafter, there might be a common immunopathogenesis involving different targets shared by these two diseases. **Figure 1** illustrates the possible mechanism. Genome-wide association studies have reported that MG involves genetic susceptibility loci simultaneously shared with autoimmune diseases (111). Accumulating evidence has demonstrated that the HLA locus may be a risk factor for comorbid autoimmune diseases in MG. EOMG is strongly associated with HLA-B8-DR3, which is associated with an increased risk of thyroid disease, SLE, and IM (101, 112). MS and MG share the common HLA risk allele DRB1*1501, contributing to the co-occurrence of the two diseases. HLA and GWAS are considered to be involved in the formation of an autoimmune environment and may explain the coexistence of these two diseases.

## 5 Conclusion

This review provides evidence of the association between MG and several comorbid autoimmune diseases. Among these concomitant autoimmune diseases, thyroid disease in autoimmune diseases and NMO in neurological autoimmune diseases are the most prevalent and well-established. Patients with MG complicated with autoimmune diseases tended to have mild clinical manifestations. In contrast, the clinical course of neurological autoimmune diseases was severe, and we observed a difference in combined neurological autoimmune diseases between the EOMG and LOMG groups. Neurological autoimmune diseases are usually combined with thymoma or thymic hyperplasia and occur most often after MG. The co-occurrence of MG and autoimmune diseases could be attributed to a similar immunological mechanisms involving different targets and a common genetic predisposition. However, the mechanisms underlying the comorbid autoimmune diseases in MG remain unclear and require further investigation. A multicenter study of patients with MG is required to confirm the conclusions of this study.

## Author contributions

All relevant articles were reviewed by two authors YZ and YH separately for considering the inclusion criteria, excluding review articles, comments/editorials, articles that were lack of relevant information. The reference lists of eligible articles were also reviewed in case of omission by BW. Controversies in data extraction between authors were confirmed by a third person, RZ. All authors contributed to the article and approved the submitted version.
